# A Comparison of medication management with separate psychotherapy to medication prescribing with psychotherapy

**DOI:** 10.1192/j.eurpsy.2024.1457

**Published:** 2024-08-27

**Authors:** L. Mehl-Madrona, B. Mainguy

**Affiliations:** ^1^Native Studies; ^2^Intermedia, University of Maine, Orono, United States

## Abstract

**Introduction:**

Little research has been conducted on outcomes in mental health care by intensity of level of service.  Mental health care has evolved in the United States to psychiatrists or psychiatric nurse-practitioners overseeing medications in 15-minute appointments while non-physicians provide the psychotherapy.

**Objectives:**

We wished to compare these two models when one psychiatrist worked in two settings, providing the medication management alone model in one setting and the medication + psychotherapy model in the second setting.

**Methods:**

All patients were seen by the same psychiatrist at (1) a community mental health center (CMHC) and (2) a private practice (PP) providing services to the same type of patients over 2 years. Patients were assessed with the My Medical Outcomes Profile (MYMOP2) and the Brief Psychiatric Rating Scale (BPRS). In the CMHC, patients were seen for a 15 – minute visits every 1 to 3 months. Patients were offered psychotherapy, ranging from 1/2 hour monthly to 1 hour every other week. Some patients received weekly psychotherapy due to an interest by the clinician. In the PP, patients were seen every 1 to 4 weeks by the psychiatrist who also provided psychotherapy when that was desired. Visits ranged from 15 to 75 minutes. Other practitioners could have also provided psychotherapy. Analysis was conducted for patients who completed at least four outcome ratings. Multi-level modeling techniques as implemented in SPSS were used to determine if patients improved over time.

**Results:**

There were no differences in age, socioeconomic status, type of insurance, and type of diagnosis among the two groups. Follow-up occurred for two years. On average, no improvement occurred in outcome measurements in the CMHC setting while statistically significant improvement occurred in the PP setting. The cost of care was statistically significantly greater in the CMHC setting, due to the facility fees billed and collected for each patient (and approved by the government) of $176 additional per visit.

**Conclusions:**

Further work can be done on establishing minimal levels of service delivery that can produce improvement for large populations in community settings. Since it is unlikely that we can generate control groups of no treatment, perhaps analyses like this one, comparing treatment models, can establish a benchmark from which we can understand the necessary level of treatment. The PP setting may have afforded more attention for patients than the CMHC setting, though at a lower cost to the government. The psychiatrist believed that he wanted patients to improve equally in both settings, but he could have been more enthusiastic in the setting in which he also did psychotherapy and therefore had better relationships with patients. On the other hand, this may be the point – better relationships with patients may be associated with better outcomes.

**Disclosure of Interest:**

None Declared

